# Educational outcomes for children and young people with cancer: study protocol for a population-based cohort study using linked education and hospital data from England

**DOI:** 10.1136/bmjopen-2025-109410

**Published:** 2025-11-24

**Authors:** Selina Nath, Tony Stone, Joseph Lam, Richard G Feltbower, Darren Hargrave, Martin G McCabe, Harry Brown, Lauren Cara Louise Hickinbottom, Kella Jackson, Lewis Paget, Tilly Stanley, Phoebe Skye Watts, Katie Harron

**Affiliations:** 1 UCL Great Ormond Street Institute of Child Health, University College London, London, UK; 2 University of Leeds, Leeds, UK; 3 Great Ormond Street Hospital for Children, London, UK; 4 Division of Cancer Sciences, University of Manchester, Manchester, UK

**Keywords:** Cancer, Education, Medical, EPIDEMIOLOGY, Schools

## Abstract

**ABSTRACT:**

**Introduction:**

Childhood cancer survivors (CCSs) experience educational disruptions during and following treatment, yet robust, longitudinal evidence on educational performance remains limited. We will investigate differences in educational outcomes between CCSs and non-cancer peers during primary and secondary school. We will also explore how sociodemographic factors and age at diagnosis contribute to potential differences in General Certificate of Secondary Education (GCSE) examinations, a critical indicator of future academic and employment prospects.

**Methods and analysis:**

We will use the Education and Child Health Insights from Linked Data (ECHILD) to capture linked health and education data for children born in National Health Service (NHS)-funded hospitals in England. We will generate birth cohorts spanning September 1997 to August 2015 (estimated sample size: ~10 million), formed of pupils expected to have undertaken national curriculum assessments between academic years 2004/2005 and 2021/2022 including Key Stage (KS) 1, 2 and 4, corresponding to ages 7, 11 and 16 respectively. Cancer diagnosis will be identified from inpatient hospital records, using International Classification of Diseases, 10th Revision codes (ICD-10). We will investigate differences between CCS and their non-cancer peers in terms of their sociodemographic characteristics and describe trends in educational performances at all KSs, recorded Special Educational Needs and Disabilities (SEND) and school absences. Differences in KS4 (GCSE) performances between CCS and non-cancer peers will be quantified, according to and accounting for geographic region, sex, deprivation, ethnicity and birth characteristics. To assess whether cancer diagnosis disrupts academic trajectories, we will restrict analysis to those with KS2 attainment data and investigate KS4 performance. We will finally explore the influence of age at diagnosis on educational performance at KS4.

**Ethics and dissemination:**

Ethics approval was granted by NHS Health Research Authority Research Ethics Committee (20/EE/0180). Findings will be shared with academics, policymakers, children and families affected by childhood cancer, and published in journals. Code/metadata will be shared on ECHILD GitHub repository.

**STRENGTHS AND LIMITATIONS OF THIS STUDY**

- We use linked education and health data covering all births in National Health Service (NHS) hospitals in England.
- The large sample size enables analyses stratified by cancer subgroups and sociodemographic factors, including up to 16 years of follow-up from birth.
- Cancer diagnoses are identified from inpatient hospital records only (International Classification of Diseases, 10th Revision (ICD-10) codes), which may miss cases treated exclusively in outpatient settings.
- Educational data are limited to state-funded schools and do not include children educated in private or alternative settings.
- The Education and Child Health Insights from Linked Data (ECHILD) dataset lacks detailed clinical information on cancer treatment and primary care interactions.

## Introduction

### Background literature

 Childhood cancer, although rare, remains the leading cause of mortality among children aged 1 to 9 years.^1 2^ In England, approximately 1450 children (aged 0–14 years) and 668 young people (aged 15–19 years) are diagnosed annually,^3^ with the most prevalent cancers during childhood being haematological malignancies (including leukaemia and lymphoma) and cancers of the central nervous system (CNS).^4^ Advancements in treatment over the past decade have steadily increased survival rates, with over 80% of children living at least 5 years post-diagnosis.^5^ Despite these advancements, cancer and its prolonged treatments (often extending over months or years) may have short and long-term physical, psychological and social effects on survivors.^6^,^10^ As the population of Childhood Cancer Survivors (CCSs) grows, there is a pressing need to identify key factors that shape their life trajectories and improve quality of life. The term ‘childhood cancer survivor’ is used in alignment with existing literature and insights shared from our patient and public involvement (PPI) experts by lived experience advisory groups for this study. However, we acknowledge this terminology may not resonate with all individuals who have experienced cancer during childhood.^11 12^ Some may prefer to emphasise thriving beyond their diagnosis rather than the notion of survival, while others may identify differently based on their unique experiences and personal journeys. Additionally, although the term encompasses individuals diagnosed between birth and 18 years of age, we acknowledge that those in mid-to-late adolescence may identify as young people rather than children.

Educational outcomes are key indicators of future employment, income, progression and long-term quality of life.^10 13^ Childhood cancer may adversely affect children’s educational achievements due to treatment time and side effects, prolonged school absences, disrupted peer relationships and insufficient collaboration between schools, healthcare providers and families.^14 15^ In the literature, educational outcomes of specific interest have been operationalised as attainment level and performance. Previous studies have predominantly focused on attainment level, defined as the highest level of educational level completed.^16^,^19^ Fewer studies have looked at children’s performance, defined as the marks or gradings obtained from an assessment.^17^ Performance throughout both primary and secondary school lays an important foundation for learning,^20 21^ and General Certificate of Secondary Education (GCSE) grades, especially in mathematics, are associated with higher future income and employment prospects.^22^ Additional factors that may influence educational outcomes include educational support (special educational needs and disabilities; SEND) and school absenteeism.

Systematic reviews and meta-analyses on educational attainment literature have consistently reported survivors of CNS cancers to have lower educational attainment compared with non-cancer peers.^16^,^19^ However, findings from studies that group all types of cancer survivors together show inconsistent and conflicting findings.^16 17^ This is likely due to the inclusion of broad studies with varied designs, populations and outcomes.^23^ Many studies were not population-based, had small sample sizes and lacked statistical power to detect differences, particularly given the rarity of childhood cancer and the diagnostic subgroups.^16^ Meta-analyses also showed inconsistencies. Gummersall *et al*^18^ reported that survivors were less likely to complete secondary school and university. Similarly, Saatci *et al* ^19^ found that CCSs were more likely to have only completed compulsory education (usually secondary school in most countries) and less likely to have completed university. There were no differences between CCSs and their peers in non-compulsory education before university, such as college-level education. Hernádfői *et al* ^24^ demonstrated a lack of evidence for differences in educational attainment across different levels; secondary education, college or bachelor’s degree, and postgraduate studies. Variability in meta-analysis findings may reflect methodological heterogeneity. A meta-analysis^18^ comparing pooled estimates from self-report and registry studies found that studies relying on self-reported data did not identify differences in educational attainment between CCSs and their peers. In contrast, registry-based studies showed CCSs were less likely to complete secondary education than their peers.

Registry studies conducted in Canada, Denmark, Finland, Italy, Norway and Sweden using national (or province) health and education records have consistently shown lower educational attainment among CCSs compared with their peers.^25^,^33^ A recent study^34^ combining data from Denmark, Finland and Sweden found that CCSs had a greater risk of not completing secondary school, compared with peers and siblings, with risks being greatest for CNS tumour survivors and weakest when survivors of all types of cancer were grouped together. However, these countries have different education systems to that found in England. Research in Scotland^35^ has also shown that children with a history of cancer had higher odds of requiring special educational support, increased absenteeism and lower academic attainment, particularly those with haematological or CNS cancers.

A gap in the literature is the lack of longitudinal analyses of educational outcomes over time. The few studies that have explored pupil performances have often focused on single time-point assessments.^827 36^,^38^ Overall, previous research has been limited by restricted access to multi-timepoint educational data and health records, preventing a full understanding of long-term academic trajectories. Another gap relates to inconsistencies in findings on how age at diagnosis affects educational outcomes. Some research indicates that being diagnosed before age 10 leads to poorer long-term academic outcomes, compared with peers without cancer,^18^ due to treatment-related neurodevelopmental effects on the developing brain during early childhood.^39 40^ Other studies suggest that diagnosis and treatment during adolescence (ages 10–19) may impact prefrontal cortex development, which plays a crucial role in executive functioning and learning.^41 42^ Additionally, diagnosis near assessment time point may impact on performance due to school absences for treatment and recovery being in close proximity to examinations.^14 43^

This study will examine the impact of age and the timing of diagnosis in relation to educational performances. Findings could inform school-based interventions to better support children and young people (CYP) diagnosed with cancer and improve their educational performance during compulsory education, subsequently impacting on their long-term outcomes beyond secondary school.

### Aim and objectives

The overarching aim of this study is to investigate educational performances of CCSs and evaluate how this differs according to sociodemographic characteristics and age at diagnosis, compared with non-cancer peers. We will also investigate how SEND and school absences vary between CCSs compared with non-cancer peers. Analyses will be stratified by cancer type (leukaemia, brain/CNS, lymphomas and any other cancers).

#### Objectives

##### Objective 1

To describe, for all cancers categorised together and stratified by cancer type, differences in educational outcomes between CCS and non-cancer peers for:

- The proportion of children sitting examinations and proportion who did not complete examinations as expected at KS1 (age 7), KS2 (age 11) and KS4 (GCSEs, age 16).
  Of those who did sit examinations:
- The proportion of children achieving the expected levels of the National Curriculum in line with Department for Education standards, and performances (standardised test scores), at KS1, KS2 and KS4.
- The proportion of children with recorded SEND or an Education, Health and Care Plan (EHCP) by the end of each KS.
- The total number of absences and proportion of children with persistent absences (>10% of missed sessions within a term) during the final year of each KS.

##### Objective 2

To explore factors contributing to differences in primary outcome KS4 GCSEs, between CCS and non-cancer peers by:

- Quantifying the extent to which any differences in KS4 GCSE results between CCS and non-cancer peers are explained by sex, ethnicity, deprivation and region of England.
- Evaluating the extent to which differences between CCS and non-cancer peers differ according to sex, ethnicity, deprivation and region of England (through interaction terms), to investigate potential inequalities.
- Exploring whether cancer diagnosis disrupts educational performance trajectories, by comparing pre-morbid KS2 performance with post-diagnosis KS4 GCSE performance (restricting our cohort to those with complete data at KS2 and KS4 GCSE assessments, where diagnosis occurs between the two KSs). We will identify children whose performance declined, remained stable or improved between KS2 and KS4, to explore patterns of recovery and persistent disadvantage.

##### Objective 3

To investigate the sensitivity of KS4 GCSE outcomes to age at diagnosis by:

- Evaluating differences in KS4 GCSE outcomes based on two categorical ages at diagnosis groupings.
  - Academically aligned categories, structured to reflect KSs: No diagnosis, diagnosis at age 0–7 years (ending KS1), 8–11 years (ending KS2) and 12–16 years (ending KS4)
  - Standard childhood cancer reporting categories, reflecting developmental stages and healthcare classifications: No diagnosis, diagnosis at age 0–4 (infancy and early childhood), 5–9 years (middle childhood), 10–14 years (early adolescence) and 15–16 years (late adolescence, overlapping with Teenagers and Young Adults classification).
  - We will additionally report on those diagnosed <1 years old, which represents a period of higher cancer incidence and unique developmental vulnerability.
- Evaluating differences in KS4 GCSE outcomes according to a continuous variable for age at diagnosis (excluding those with no cancer diagnosis).

## Methods and analysis

### Study design

This is an observational population-based cohort study using linked administrative data from health and educational databases in England (Education and Child Health Insights from Linked Data (ECHILD)). We will develop birth cohorts of CYP, with longitudinal follow-up on education and health. The study commenced in July 2024 and is scheduled to conclude by July 2026.

### Data sources and linkage

ECHILD contains linked, longitudinal records from routinely collected health and education data.^44^ Health-related data is derived from Hospital Episode Statistics (HES), a national database consisting of all information collected in the National Health Service (NHS) acute hospital care (including inpatient, outpatient, emergency services and diagnosis) and mortality data.^45^ Using HES, children can be followed from their birth admission throughout all subsequent NHS hospital contacts.^46^ In England, ~97% of children are born in NHS hospitals; however, HES does not include data from births in private hospitals or home settings.

Education data are available from the National Pupil Database (NPD), which captures comprehensive information on CYP attending state-funded schools in England.^47^ This includes data on assessments conducted at ages 5, 7, 11, 16 and 18 years, SEND and school absences. In England, full-time education is compulsory from ages 5 to 16, with the participation extended to age 18 between years 2013–2015. Primary school education begins at age five and transitions to secondary school education at age 11. Pupils follow the National Curriculum, which is divided into phases called KS: assessments occur at age 7 (KS1), 11 (KS2) and 14 (KS3), with national examinations (GCSEs) administered at age 16 (KS4) and assessments such as A-levels and other post-16 qualifications at age 18 (KS5). The database also records school-level characteristics, such as school type.

ECHILD does not capture data on CYP educated in the private sector (approximately 7% of pupils in England annually). Within ECHILD, ~99% of pupils in the NPD have been linked to their NHS ID. However, the NHS national data opt-out was applied to ECHILD, affecting approximately 5.8% of identified linkages. For further details on linkage methodology, see the ECHILD user guide.^48^

### Study population and follow-up

We will develop birth cohorts of children and young people using ECHILD. The study population will include all children born in NHS hospitals in England between first September 1997 and 31^st^ August 2022 (HES birth cohort, linked to NPD (estimated sample size~10 million). Estimates are derived from previous ECHILD linkage reports published elsewhere.^48 49^

Educational outcomes will be obtained by linking the HES birth cohort to records in the NPD for children assessed at school during the academic years 2004/05 to 2021/22. We will extract relevant education and health data, including attainment and performance in KS assessments. Cancer diagnoses will be identified from HES Admitted Patient Care (APC) data, capturing the earliest diagnosis (if any) across four broad cancer categories: Leukaemia, Brain/CNS, Lymphomas and any other cancers. Death registration data from the Office for National Statistics (ONS), including date and cause of death, will be used to supplement diagnostic records in HES APC. To validate cancer diagnoses, we will cross-reference these with publicly accessible cancer incidence figures from NHS England’s Cancer Data (National Cancer Registration and Analysis Service).^2^

**Figure 1:**
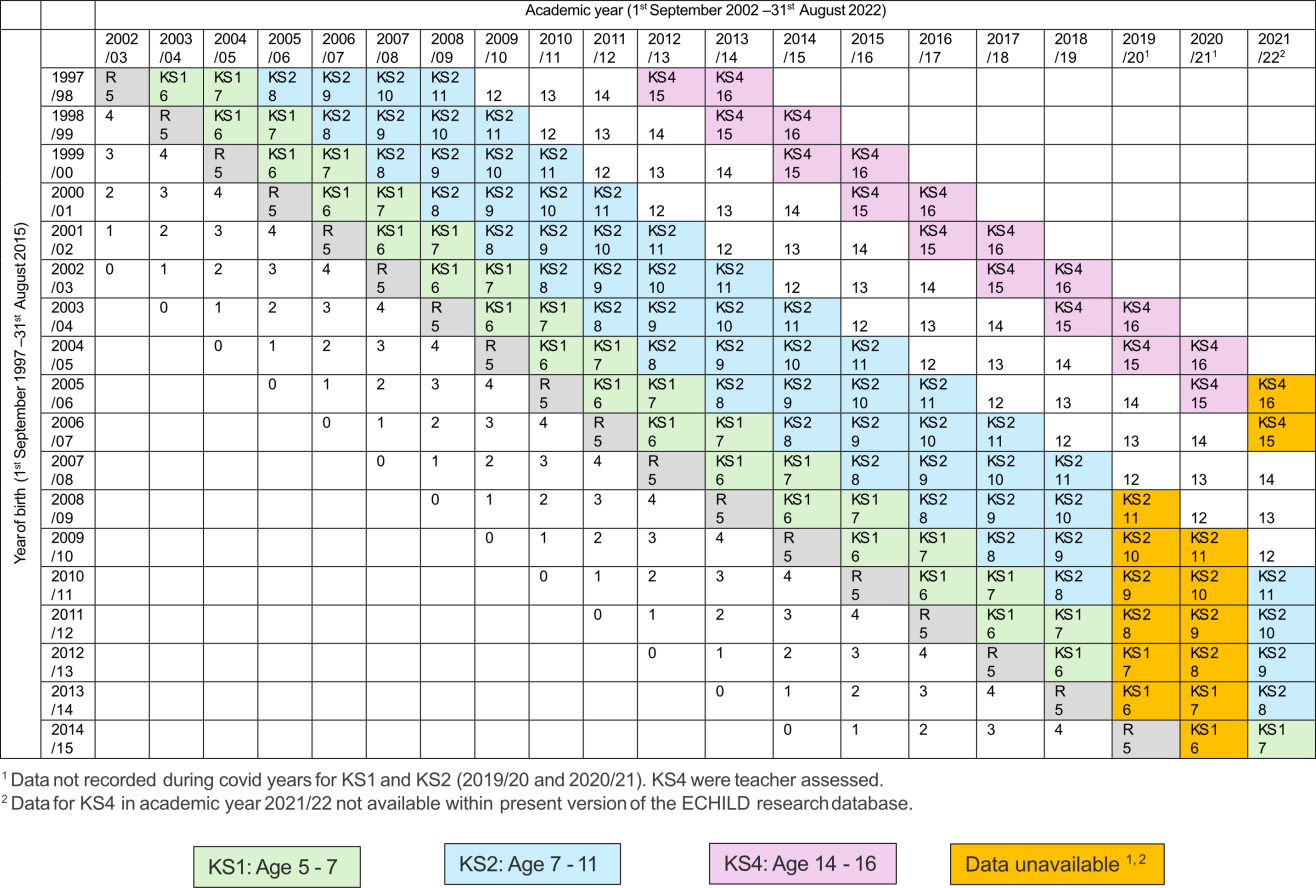
Expected age during primary and secondary school by year of birth and follow-up years in education. ECHILD, Education and Child Health Insights from Linked Data; KS, key stage; R, reception.

We will create three cohorts, each capturing a differing length of follow-up (See figures1  2). Each sequential cohort will include a longer follow-up period but smaller population sample. Cohort 1 will include births between the academic years September 1997 to August 2015 and capture KS1 outcomes at age 7, excluding those born between September 2012 to August 2014 (due to their KS1 outcomes not being captured during the COVID pandemic). Cohort 2 will include births between September 1997 and August 2011 and capture KS2 outcomes at age 11, excluding those born between September 2008 and August 2010 (due to COVID). Cohort 3 will include births between September 1997 and August 2005 and capture KS4 outcomes at age 16.

**Figure 2:**
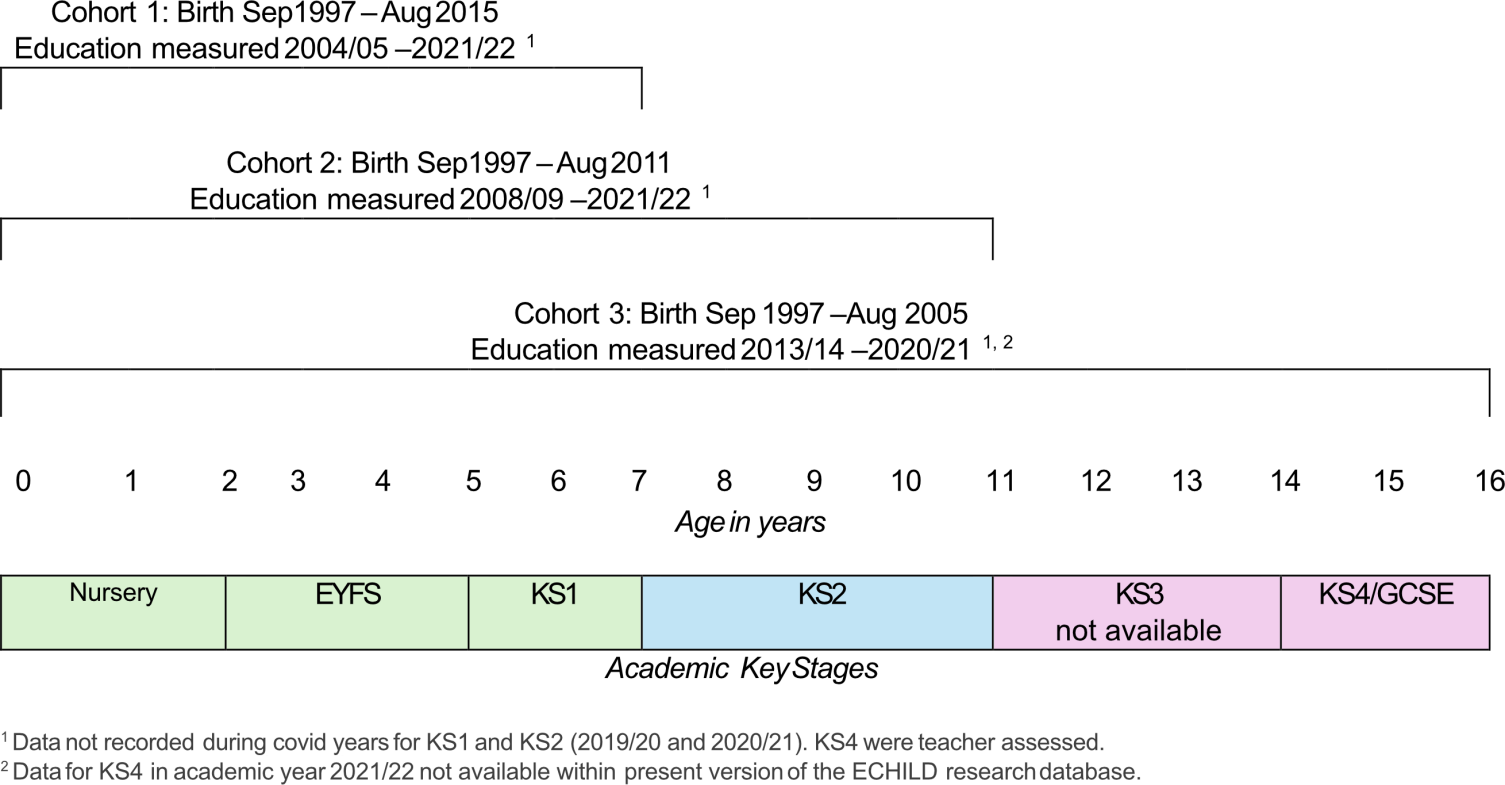
Cohort academic birth years, ages and education key stages. ECHILD, Education and Child Health Insights from Linked Data; EYFS, Early Years Foundation Stage; GCSE, General Certificate of Secondary Education; KS, key stage.

### Key study measures

#### Exposure: childhood cancer

Childhood cancer diagnoses will be identified using International Classification of Diseases, 10th Revision (ICD-10) codes as recorded in HES APC data.^50 51^ Most children diagnosed with cancers will be admitted to hospital at some point during their diagnosis and treatment. Groups will include four broad diagnostic cancer types, three with greatest incidence in childhood, and all other cancers combined. The three broad diagnostic cancer groups with greatest incidence are Leukaemia, CNS, Lymphomas and any other cancers, accounting for, respectively, 32%, 24% and 12% of all childhood (0–14 years) cancers recorded in England in the period 1997 to 2016.^3^ Children diagnosed with cancer will be compared with peers with no recorded diagnosis of cancer prior to the July following the relevant KS outcome assessment. Age at diagnosis will be derived by using HES birth date and diagnosis date and categorised using two age at diagnosis groupings. Academically aligned categories will be structured to reflect UK KSs: No diagnosis, diagnosis at age 0–7, age 8–11 and age 12–16, to correspond to ending at academic years of school for year 2 (6/7, KS1), year 6 (10/11, KS2) and year 11 (15/16, KS4). Standard childhood cancer reporting categories will also be used, to reflect developmental stages and healthcare classifications: No diagnosis, diagnosis at age 0–4 (infancy and early childhood), age 5–9 (middle childhood), age 10–14 years (early adolescence) and 15–16 years (late adolescence, overlapping with Teenagers and Young Adults classification). We will additionally report on those diagnosed <1 years old, which represents a period of higher cancer incidence and unique developmental vulnerability.

#### Outcomes: educational outcomes

We will evaluate educational performances at each KS. For KS1 and KS2, using a binary outcome (those who reached expected levels of the National Curriculum as defined with Department of Education standards), and a continuous standardised point score to measure performance.^52^ Scores will be standardised within years, to make fair comparisons across years. Data on assessments at KS3 are not available in ECHILD. For KS4, we will assess the number and proportion of children achieving 5 A*- Cs (9 - 4 grades in reformed GCSEs), including English and Maths, and standardised point scores in English and Maths GCSE exams.^53^ We will evaluate the number and proportion of children sitting examinations (ie,appearing in the school Census and sitting the exam) and those who did not complete the examinations as expected (ie, appearing in the Census but not having an assessment record) at KS1 (age 7), KS2 (age 11) and KS4 (GCSEs, age 16).

We will also evaluate the number and proportion with recorded SEND^16^,^19^ or an EHCP. SEND provision will be grouped in the following descending hierarchy: 1. No SEND provision, 2. SEND support in mainstream school, 3. EHCP in mainstream school, 4. Enrolment in specialist provision. School absences will be defined as: 1. pupils who miss 10% or more of their possible sessions (persistent absence), 2. authorised/unauthorised absences for doctor/dentist appointments or illness and 3. Authorised/unauthorised absences for all other reasons than medical. Absence data is available from 2005/06 and reason for absence is available from 2006/07. Data were not recorded during academic years 2019/20 and 2020/21 for KS1 outcomes, KS2 outcomes and school absences due to the covid pandemic. KS4 outcomes data during these years were teacher assessed, rather than based on formal examination marks. KS4 data are not available for 2021/22 within the current version of ECHILD.

#### Additional covariates of interest

We will derive a set of sociodemographic characteristics including year of birth, sex, ethnic group, region of England, and area-level deprivation (quintile of the Index of Multiple Deprivation; IMD). Infant birth characteristics will include gestational age at birth defined as very preterm (<32 weeks), moderate preterm (32–33 weeks), late preterm (34–36 weeks), and term (≥37 weeks), and congenital anomalies, identified using an International Classification of Diseases version 10 (ICD-10) code list.^51^ We will also flag individuals with Down syndrome, neurofibromatosis or tuberous sclerosis (recorded in hospital records within the year from birth).^34^

### Statistical analysis plan

#### Cohort description

We will use a CONSORT-style flowchart to illustrate the linkage process between HES and NPD records, and to show the inclusion and exclusion of children at each stage leading to the formation of the three cohorts for analysis. For each cohort, we will compare the characteristics of children who were linked with those who were not, as well as children with and without available data at each KS. We will report on follow-up completeness and attrition for children diagnosed with cancer and those without. Baseline sociodemographic and clinical characteristics will be described for children with and without a cancer diagnosis across the cohorts. Differences will be visualised graphically and evaluated using standardised mean differences to assess the size of any differences between groups. We will additionally report age at cancer diagnosis for affected children within the cohort.

##### Objective 1

We will quantify the proportion of children in each group with each binary outcome and describe mean standardised scores across groups for continuous outcomes. We will model the proportion of children achieving expected levels at each KS using generalised linear regression to generate relative risks and 95% CIs. We will use linear regression to estimate the difference in standardised scores at each KS between groups. Models will account for clustering of pupils within schools using robust standard errors.

##### Objective 2

We will adopt a causal inference framework to guide analysis. Building on our model for KS4 outcomes (Cohort 3) from Objective 1, we will evaluate the extent to which differences in outcomes between groups are explained by key covariates. Key confounders will be identified a priori using directed acyclic graphs (DAGs) based on a combination of inputs by experts with lived experience, clinical/epidemiological knowledge and existing literature. This approach supports transparency in assumptions and helps avoid inappropriate adjustment.^54^ While we aim to strengthen causal interpretation, we recognise the inherent limitations of observational data and will interpret our findings with appropriate caution.

To determine whether the effect of having cancer differs according to sociodemographic group, we will include interaction terms within the models. This will enable us to identify any inequalities in outcomes which exist according to, for example, sex, ethnicity or deprivation.

To explore whether cancer diagnosis disrupts educational performance trajectories, we will restrict our analysis to the subset of children with complete data on assessments at KS2 and KS4, who had a cancer diagnosis between the two assessment time points. We will describe the number of children achieving expected levels at KS2 who went on to either achieve expected levels at KS4 or not. Additionally, we will explore patterns of recovery and persistent disadvantage by identifying children whose performance declined, remained stable or improved between KS2 and KS4.

##### Objective 3

Building on our model for KS4 outcomes (Cohort 3) from Objective 1 and 2, we will quantify the proportion of children achieving 5A*- Cs (9 - 4 grades in reformed GCSEs), including English and Maths and compare average standardised point scores at GCSE according to categorical age at diagnosis. Relative risks for each age group will be estimated from a generalised linear regression model. For those with cancer only, we will create a separate model including continuous age at diagnosis (in years) to evaluate the effect of each individual year on outcomes.

We will further explore the effect of timing of diagnosis in relation to outcomes by restricting our cohort to children aged 0–5 at diagnosis and comparing effect sizes for KS1, KS2 and KS4.

### Missing data

We will quantify missing data in covariates and assess whether this is associated with our primary outcome (GCSE grades at KS4). For ethnicity, missing data will be categorised as unknown. Decisions on whether to use complete case analysis or multiple imputation will be taken based on levels and patterns of missingness. We will conduct sensitivity analysis where required.

### Patient and public involvement and engagement

Previously published patient and public involvement and engagement (PPIE) with CYP and their carers identified educational outcomes as a key priority for data use,^55^ and highlighted communication and involvement of CSS as a key component to public and patient trust on data use for research. Through several previous consultation works conducted within our broader child health informatics team at Great Ormond Street Institute of Child Health, University College London,^56^ acceptance was found among CYP on the use of administrative data for research, provided that they are included in the research process.

This study is conducted in consultation with a Young Person’s experts by lived experience advisory group, including those who self-identify as experts by lived experience, survivors and warriors. Members were recruited via Young Lives vs Cancer. Their input has informed the development of research priorities and will guide interpretation of findings from the ECHILD linked health and education administrative data. Engagement is structured across four meetings aligned with key project milestones. Group members are invited to contribute as co-authors on research publications.

In the first meeting (April 2025), the advisory group helped identify research priorities. Educational outcomes and inadequate support, especially within the educational systems, emerged as central concerns. Members emphasised the lasting effects of cancer diagnosis and treatment, and the difficulty of reintegrating into education. They described a system that often overlooks emotional and social well-being, focusing narrowly on physical recovery and offering few accommodations. In the second meeting (July 2025), the group inputted into our analysis plan through discussions on covariates included in the analysis which cause inequalities (see Methods section), how age at diagnosis impacted on education and understanding of what is missing in our data. This knowledge will inform the construction of a DAG and provide contextual understanding.

In a separate consultation (June 2025), we engaged with a group of parents of young children diagnosed with cancer. Their feedback echoed concerns around inadequate educational support and highlighted the isolating experience of advocating for their children’s rights to education. Parents described a prevailing expectation for children to simply ‘slot back’ into education following treatment, with limited awareness or provision for their emotional well-being. They also provided useful insights into potential sources of missing data in our cohort.

A key theme that emerged from our PPIE activities was the complexity of accessing support and navigating the education system. CYP were often expected to seamlessly resume their education post-treatment without adequate consideration of short- and long-term physical and emotional challenges. The rapidly changing and fluid nature of cancer treatment is not accommodated effectively by the current education system, such as Education and Healthcare plans. There was also reported inflexibility of exam boards and the wider education system with regards to formal examinations, which would be particularly pertinent to our research objective focusing on GCSE results. Although our dataset does not fully reflect these nuanced experiences, we are committed to integrating these insights into the interpretation of our findings to better understand the contextual realities. We also aim to embed lived experience voices in future mixed-methods research to more fully capture the narratives underlying the data.

We did not require ethics review to conduct this involvement activity as the young people and parents were advisors to the study team rather than research subjects.

## Ethics and dissemination

### Ethics

Approvals to access and to conduct statistical analyses of linked, de-identified data from HES and the NPD were obtained from NHS England (DARS-NIC-381972) and the Department for Education (DR200604.02B). Ethical approval for the ECHILD project was obtained from the NHS Health Research Authority Research Ethics Committee (REC references: 20/EE/0180 and 21/SW/0159), the National Research Ethics Service (17/LO/1494) and the UCL Great Ormond Street Institute of Child Health Joint Research and Development Office (20PE06). As the study uses de-identified, routinely collected administrative data, individual informed consent was not required and was waived by the approving ethics committees. All data were anonymised prior to access by the study team.

### Dissemination

We aim to ensure transparency, reproducibility and impact across academic, policy and public domains. Study methods and analysis code will be shared via the ECHILD GitHub repository and within the secure research environment of the Office for National Statistics (ONS). We will present preliminary findings to academic and policy audiences and engage our PPI advisory groups, young people and parents/carers, through structured consultations to share emerging results and gather input on interpretation of findings. Final outputs will include peer-reviewed articles, a funder report, and accessible materials such as infographics for non-academic audiences. The cohorts developed will support future research into long-term educational and health outcomes for CYP affected by cancer. Due to data-sharing agreements with NHS England and the Department for Education, the ECHILD database is not publicly available. Access is limited to accredited researchers via application to the ECHILD team and approval from ONS.
