# Supplementary material for: Educational outcomes for children and young people with cancer: study protocol for a population-based cohort study using linked education and hospital data from England
Source: BMJ Open. 2025 Nov 24;15(11):e109410. doi: 10.1136/bmjopen-2025-109410 (PMC12645591; doi:10.1136/bmjopen-2025-109410)
Supplement: Supplementary file 1 [file bmjopen-2025-109410.reviewer_comments.pdf]

PEER REVIEW HISTORY

BMJ Open publishes all reviews undertaken for accepted manuscripts. Reviewers are asked to complete a checklist review form and are provided with free text boxes to elaborate on their assessment. These free text comments are reproduced below.

ARTICLE DETAILS

Title (Provisional)

Educational outcomes for children and young people with cancer: study protocol for a population-based cohort study using linked education and hospital data from England.

Authors

Nath, Selina; Stone, Tony; Lam, Joseph; Feltbower, Richard; Hargrave, Darren; McCabe, Martin G; Brown, Harry; Hickinbottom, Lauren Cara Louise; Jackson, Kella; Paget, Lewis; Stanley, Tilly; Watts, Phoebe Skye; Harron, Katie

---

VERSION 1 - REVIEW

**Reviewer**	**1**
**Name**	**Doherty, Edel**
**Affiliation**	**University of Galway**
**Date**	**16-Sep-2025**
**COI**	**None**

Dear Authors,

Dear Authors,

I think your proposed study addresses a very important research question that meaningfully can inform policy in this area. I think the background, objectives, methods and analysis plan are adequately described. Clearly, this paper will add new knowledge on the educational outcomes of CCS. The strengths of the study include the longitudinal analysis, the use of linked data and the ability to compare child outcomes by age. These all add important contributions to the existing literature.

I have only two points for this paper:

1. It would be interesting to examine persistent disadvantage in terms of educational outcomes versus children whose educational outcomes recover after a period. This might help us to understand differences or mediating factors that might help educational outcomes recover. Some of the planned analysis may address this and I think it is worth exploring.

2. The authors note that they will examine causal effects but I am not sure if the proposed study can do this. Will any methods for causal inference be used in this study. The disadvantage of using linked data is that it does not capture a large number of potential confounder variables, which could limit the study's ability to identify causal pathways.

VERSION 1 - AUTHOR RESPONSE

Reviewer 1: Dear Authors,
I think your proposed study addresses a very important research question that meaningfully can inform policy in this area. I think the background, objectives, methods and analysis plan are adequately described. Clearly, this paper will add new knowledge on the educational outcomes of CCS. The strengths of the study include the longitudinal analysis, the use of linked data and the ability to compare child outcomes by age. These all add important contributions to the existing literature. I have only two points for this paper.

We were really pleased that you found our work important, recognising its contribution of new knowledge to the existing literature and its potential to meaningfully inform policy.

Thank you for your careful reading and helpful comments which we have fully addressed below:

1. It would be interesting to examine persistent disadvantage in terms of educational outcomes versus children whose educational outcomes recover after a period. This might help us to understand differences or mediating factors that might help educational outcomes recover. Some of the planned analysis may address this and I think it is worth exploring.

   Thank you for this helpful suggestion. We agree that exploring differences in recovery versus persistent disadvantage is important. We have extended our analysis plan under Objective 2c to describe changes in performance between KS2 and KS4 among children diagnosed with cancer between these stages, identifying those whose outcomes declined, remained stable, or improved. We will consider additional analyses to explore predictors of these patterns further, where data completeness allows.

   We have amended following next to account for this addition:
   "We will identify children whose performance declined, remained stable, or improved between KS2 and KS4, to explore patterns of recovery and persistent disadvantage."
   [Manuscript: page 7, paragraph 2, lines 11 - 13]
   "Additionally, we will explore patterns of recovery and persistent disadvantage by identifying children whose performance declined, remained stable, or improved between KS2 and KS4."
   [Manuscript: page 12, paragraph 4, lines 8 - 10]

2. The authors note that they will examine causal effects but I am not sure if the proposed study can do this. Will any methods for causal inference be used in this study. The disadvantage of using linked data is that it does not capture a large number of potential confounder variables, which could limit the study's ability to identify causal pathways.

   Thank you for this important comment. We agree that causal inference using linked administrative data has limitations, particularly due to unmeasured confounding. While our study is observational, we will adopt a causal inference framework to guide our analysis. Specifically, we will use directed acyclic graphs (DAGs) to identify key confounders, informed

by input from our experts by lived experience advisory group, clinicians, and existing literature. This approach supports transparency in assumptions and helps avoid inappropriate adjustment (Tennant et al., 2021). We will interpret findings cautiously and have clarified in the manuscript that causal inference is used as a framework to guide analysis, rather than to make definitive causal claims.

We have revised the manuscript text to reflect this more clearly:
"We will adopt a causal inference framework approach to guide analysis. Building on our model for KS4 outcomes (Cohort 3) from Objective 1, we will evaluate the extent to which differences in outcomes between groups are explained by key covariates. Key confounders will be identified a priori using directed acyclic graphs (DAGs) based on a combination of inputs by experts with lived experience, clinical/epidemiological knowledge and existing literature. This approach supports transparency in assumptions and helps avoid inappropriate adjustment [54]. While we aim to strengthen causal interpretation, we recognise the inherent limitations of observational data and will interpret our findings with appropriate caution."
[Manuscript: page 12, paragraph 3].
